# Peripheral primitive neuroectodermal tumors of the spine: a case report and review of the literature

**DOI:** 10.1186/s13104-016-2246-5

**Published:** 2016-09-09

**Authors:** Mouna Khmou, Abderrahmane Malihy, Najat Lamalmi, Lamia Rouas, Zaitouna Alhamany

**Affiliations:** 1 Department of Pathology, Children’s Hospital, Ibn Sina University Hospital, Ibn Sina bd Ibn Rochd, Souissi, 10100 Rabat, Morocco; 2 Faculty of Medicine and Pharmacy Rabat, University Mohammed V, Rabat, Morocco

**Keywords:** PNET, Spine, Extra-dural, Tumors, Childhood

## Abstract

**Background:**

Peripheral primitive neuroectodermal tumors are extremely rare tumors in the spine; only 18 cases of extra-dural **peripheral primitive neuroectodermal tumor** cervical region have been reported. The aim of this report is to highlight the challenges in diagnosis and management of this condition.

**Case presentation:**

We present a case of 5-year-old **Moroccan** boy, who presented with torticollis for 1 month. **Computed tomography** scan and Magnetic resonance imaging of the cervical spine revealed an extradural, dumbbell-shaped mass with extra-spinal extension at the left C1–C6 level. Multiple biopsy specimens were obtained. Histological examination revealed a highly cellular neoplasm composed of diffuse sheets of tumor cells having monomorphic, round to oval, finely vesicular nuclei. Immunohistochemical findings confirmed the diagnosis of intraspinal **peripheral primitive neuroectodermal tumor**.

**Conclusion:**

After this illustrative case, we review the literature on clinicopathological and therapeutic aspects. In practice, it is important to consider the diagnosis of peripheral **primitive neuroectodermal tumor** in children and adolescents with an apparent soft-tissue mass located in the spine.

## Background

Primitive neuroectodermal tumors (PNET) are heterogeneous group of malignant neoplasms that occur mostly in childhood and early adulthood [1]. These tumors can be classified as central PNETs (cPNET) or peripheral PNETs (pPNET) depending on the site of presentation. Primary pPNETs of the spine are extremely rare. Extradural locations are even rarer [2]. Due to their low incidence, the available epidemiology is likely unreliable, and there are currently no standard clinical guidelines outlining their management [1]. Histologically, the PNET cells exhibit a primitive, poorly differentiated morphology with varying degrees of pleomorphism and occasional evidence of neuroectodermal differentiation. The final diagnosis requires immunohistochemical analysis and cytogenetic studies to identify (11;22) (q24;q12) translocation. In this paper we present an illustrative case of a primary extradural PNET of the cervical spine, demonstrating clinical, pathological, and imaging characteristics of PNET of the spine; followed by a comprehensive review of the literature.

## Case presentation

A 5-year-old Moroccan boy presented with a 2 months history of torticollis with radiation of the pain to the left upper limb. The patient had no medical history. Physical examination revealed hypoesthesia of the upper limbs, evidence of bilateral lower extremity weakness with motor power 4/5 in distal muscular group, and hypoactive reflexes in four extremities. There was no bowel or bladder dysfunction.

**Fig. 1:**
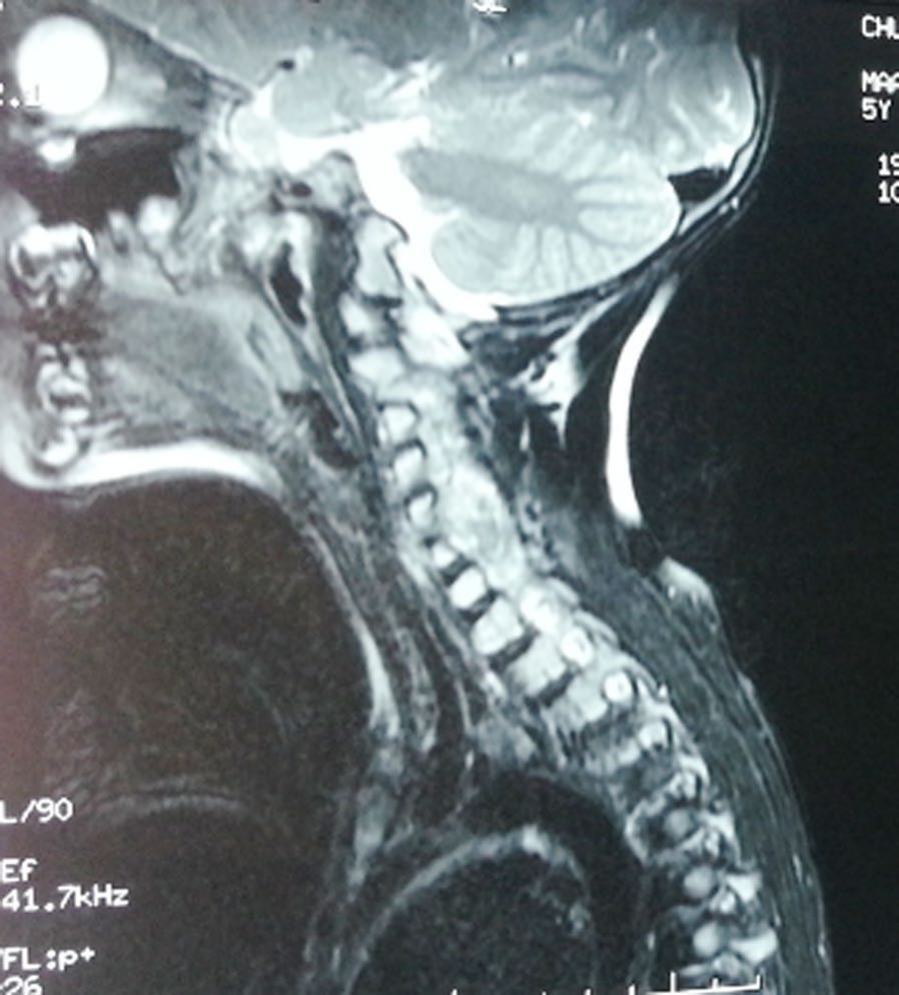
Sagittal T2 MRI showing an extradural mass observed at C1–C6 level

Cervical CT-scan and MR imaging demonstrated the presence of a 75 × 36 × 24 mm extradural mass, with extra-spinal extension at the left C1–C6 level (Fig. 1). This dumbbell-shaped mass had heterogeneous signal intensity, calcifications and homogeneous intermediate post-contrast enhancement following gadolinium administration.

**Fig. 2:**
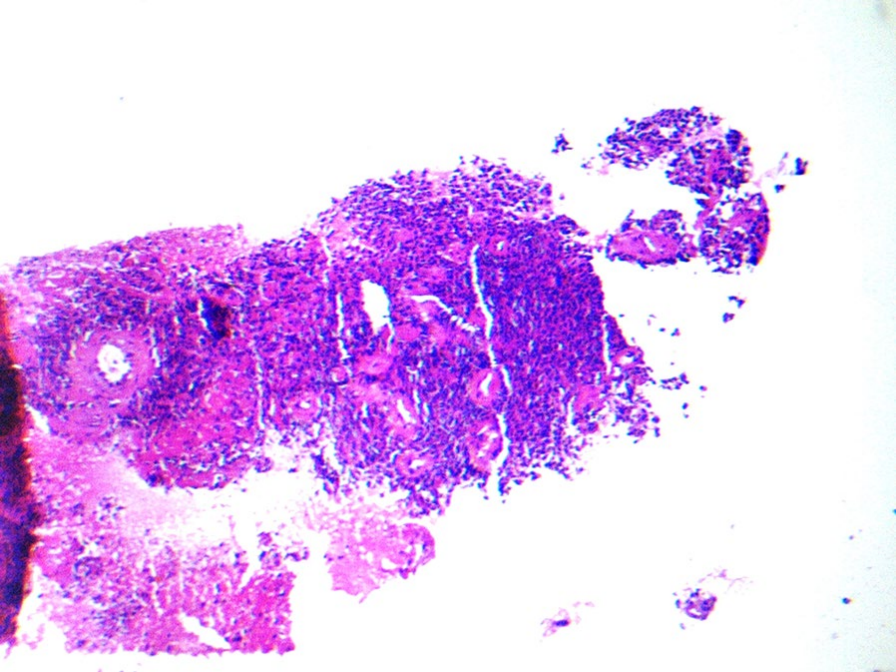
Diffuse sheets of small, round, blue cells (HE)

**Fig. 3:**
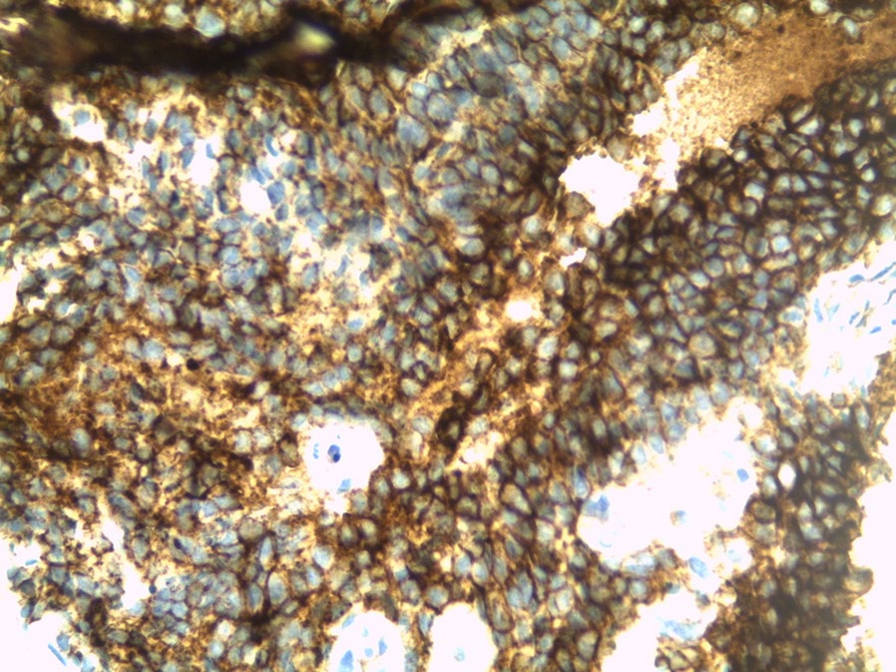
Tumor cells show diffuse membranous positivity for CD-99

No other cord lesion and no osseous abnormality of the spine were found. A biopsy was done. Microscopic sections showed a highly cellular neoplasm composed of diffuse sheets of tumor cells having monomorphic, round to oval, finely vesicular nuclei, occasional nucleoli, and scanty cytoplasm. Large areas of necrosis were seen (Fig. 2). Immunohistochemical analysis showed positivity for CD99 (Fig. 3) and S-100 protein, and negativity for synaptophysin, chromogranin, desmin, myogenin and CD56; favoring the diagnosis of PNET. After six courses of chemotherapy, cervical CT scan demonstrated complete regression of the mass. Our patient has remained in remission at 8 months follow-up.

## Discussion

Primitive neuroectodermal tumors are a group of malignant neoplasms, arising from pluripotent neural crest cells [2]. Although PNET is the second malignant neoplasm in childhood, the spinal cord, as primary site for pPNET, is relatively rare in all age groups, and information concerning these tumors in the medical literature is limited to single case reports.

Close to 107 cases of primary spinal PNET have been reported in the literature with age ranging from 4 months to 70 years, but only 18 cases of extra-dural PNET in cervical region have been reported (Table 1). In fact, the incidence of primary intraspinal PNET in lumbar region is twice as much as in thoracic and cervical regions [3], and it has been reported to have an adult onset and a male predominance [18]. Clinical presentation depends on the affected site and the degree of tumor invasion, with no pathognomonic symptom [4]. Few reports have investigated the imaging characteristics of PNET of the spine [19]. Diagnostic imaging studies should include computerized tomography (CT) and magnetic resonance imaging (MRI), but radiographic findings vary from patient to patient and are generally not helpful in differentiating PNET from other primary spinal [1]. Definitive diagnosis of PNET can only be made after tumor tissue is obtained from a limited biopsy or a radical resection specimen. Histological analysis alone is not sufficient for diagnosis; immunohistochemistry is required [1]. Microscopically, these tumors consist of small uniform round cells with rounded nuclei, fine chromatin, and an eosinophilic scanty cytoplasm. The term “Ewing’s sarcoma” is reserved for undifferentiated neuroectodermal tumors, while “PNET” is used for differentiated tumors with Homer Wright rosettes [4]. Immunohistochemically, tumor cells are positive for CD99 and HBA71, which are products of the MIC2 gene. Immunoreactivity for synaptophysin, NSE, S100 and neurofilament, indicating neuroectodermal differentiation [4]. Cytogenetic analysis detect the presence of a reciprocal translocation t (11;22) (q24;q12), it is important to confirm the diagnosis in certain cases [2]. The differential diagnosis includes central PNET, eosinophilic granuloma, malignant meningioma, rhabdomyosarcoma, neuroblastoma, and lymphoma [4, 6]. Due to the rarity of cases, specific guidelines in the management of intraspinal PNET have not yet been devised. Surgery has been considered as the initial step in the management because patients may present with neurologic deficit [5]. In recent years, chemotherapy has been showing encouraging results for the treatment of Ppnet. An induction chemotherapy tends to reduce the tumor size, as well as to control systemic disease, prior to any total excision of tumor [14, 20]. The regimen includes multiple agents such as doxorubicin, vincristine, and cyclophosphamide, alternating with ifosfamide and etoposide. Radiation therapy is commonly used as an adjuvant treatment for residual disease [4].

**Table 1:** Summary of the cases of extradural PNET in cervical spine reported in the literature

Authors	year	Age/sex	Location of tumor	Treatment
Kennedy et al. [5]	2000	24 y/M	C1–C5/extradural	STR/RT/CT
Shin et al. [6]	2001	38 y/M	C5–C7/extradural	STR/CT
Shin et al. [6]	2001	22 y/F	C7–T1/extradural	STR/CT
Mukhopadhyay et al. [7]	2001	29 y/F	C3–C5/extradural	STR/RT/CT
Mukhopadhyay et al. [7]	2001	13 y/M	C3–C5/extradural	STR/RT/CT
Kogawa et al. [8]	2004	7 y/F	C2–C4/extradural	STR/RT/CT
Ozturk et al. [9]	2007	18 y/M	C6–T1/extradural	TTR/CT
Bozkurt et al. [10]	2007	28 y/M	C3–C5/extradural	TTR/RT/CT
Erkutlu et al. [11]	2007	7 y/M	C5–T1/extradural	TTR/RT/CT
Duan et al. [12]	2010	14 y/M	C2–C4/extradural	STR/RT/CT
Sharafuddin et al. [13]	1992	21 y/F	C4–5 extradural	TTR/CT
Kumar et al. [14]	2000	12 y/F	C7–T4 extradural	Biopsy/RT
Izycka–Swieszewska et al. [15]	2001	13 y/F	C7–T11 extradural	Biopsy/CT/RT
Mukhopadhyay et al. [7]	2001	29 y/F	C3–5 extradural	STR/CT/RT
Mukhopadhyay et al. [7]	2001	13 y/M	C3–5 extradural	STR/CT/RT
Virani et al. [16]	2002	5 y/M	C7–T1 extradural	TTR/RT
Kumar et al. [17]	2007	8 y/M	C2–4 extradural	TTR
Gustavo et al. [4]	2012	22y/F	C1–C2 extradural	TTR/RT/CT
Our present case	2014	5y/M	C1–C6	Biopsy/CT/RT

*CT* Chemotherapy, *F* Female, *M* Male, *RT* Radiotherapy, *STR* Sub-total tumor resection, *TTR* Total tumor resection, *y* year

Due to the aggressiveness of these tumors, the prognosis is still poor; disease-free survival is approximately 45 % at 7 years [4].

## Conclusions

Primary spinal extradural PNET is very rare and occurs predominantly in children and adolescents. Lacking clinical and radiographic specificity, those tumors should be included in the differential diagnosis of extradural mass lesions. In order to confirm the diagnosis, histopathology and immunohistochemistry analysis are mandatory. The presence of the t (11; 22) (q24, q12) chromosomal translocation is supporting the diagnosis of pPNET. Due to limited evidence regarding the therapeutic aspects of these tumors, no definite protocol can be formulated for their treatment, but resection, radiotherapy, and multipotent chemotherapy are the preferred treatment options for these patients. Clinical outcomes still need to be evaluated in prospective trials.

## Abbreviations

PNET: peripheral primitive neuroectodermal tumors
CT scan: computerized tomography scan
